# Morphological evolution of self-deposition Bi_2_Se_3_ nanosheets by oxygen plasma treatment

**DOI:** 10.1038/srep22191

**Published:** 2016-02-29

**Authors:** Guozhi Jia, Zengna Wu, Peng Wang, Jianghong Yao, Kai Chang

**Affiliations:** 1 Tianjin Chengjian University, Tianjin 300384, China; 2 Institute of Semiconductors, Chinese Academy of Sciences, P.O. Box 912, Beijing 100083, China; 3 Key Laboratory of Weak-Light Nonlinear Photonics, Ministry of Education, School of Physics and TEDA Applied Physics School, Nankai University, Tianjin 300457, P.R. China 300457, China

## Abstract

Bi_2_Se_3_ nanosheets were successfully synthesized by a microwave-assisted approach in the presence of polyvinylpyrroli done at a temperature of 180 °C for 2 h. The thin film was prepared on a silicon wafer via a self-deposition process in a Bi_2_Se_3_ nanosheet ink solution using the evaporation-induced self-assembly method. The structure and morphology of the obtained products were characterized by X-ray diffraction, scanning electron microscopy (SEM), x-ray photoelectron spectroscopy, and Raman spectroscopy. The highly uniform Bi_2_Se_3_ particles could be formed by controlling the oxygen plasma treatment time. After the plasma pretreatment from 10 to 20 s, the surface of Bi_2_Se_3_ film evolved from the worm-like structure to particles. The highly uniform thin film was formed on further increasing the plasma treatment time, which is consistent with the observed SEM results. Several important processes can result in the morphological evolution of Bi_2_Se_3_ nanosheets: (1) formation of Bi_2_Se_3_ oxide layer; (2) self-assembly of oxide nanoparticles under the action of high-energy oxygen plasma; and (3) electrostatic interaction and etching mechanism.

Two-dimensional (2D) layered materials have attracted considerable attention in recent years because of their potential applications in electric and optoelectronic devices^1^,^2^,^3^,^4^. The morphology, thickness, and microstructure of nanomaterials have important effects on their transport and optical properties. Layered bismuth selenide (Bi_2_Se_3_), an important narrow band gap semiconductor, possesses topologically protected chiral metallic surface states and an insulating bulk^5^,^6^,^7^,^8^,^9^,^10^. Bi_2_Se_3_ has a rhombohedral structure consisting of hexagonal close-packed atomic layers periodically arranged along the c-axis^11^. The neighboring quintuple layers are bound by the weak van der Waals forces. Compared with Bi_2_Se_3_ bulk material, its nanostructures remain relatively unexplored. A number of Bi_2_Se_3_ nanostructures have been successfully synthesized using various methods for different applications. These methods are usually termed as “top-down” and “bottom-up” methods^12^,^13^. Nanomaterial ink has emerged as a low-cost alternative method for the fabrication of many functional films^14^,^15^,^16^. Lin *et al.* report the use of 2D colloidal Bi_2_Se_3_ nanoplates as an ink material to obtain high-performance electronic thin films via a solution assembly method^17^. It is an easy and common technology to realize 2D nanoplates as a building block for Bi_2_Se_3_ thin films on any substrates for the application in electronic and optoelectronic devices.

Recently, the effect of surface oxide layers on Bi_2_Se_3_ electronic characteristics has been investigated^18^. In fact, the formation of surface oxide layer is inevitable during the preparation of materials and devices. The intentionally growing oxide insulator layers can significantly improve the photoelectric characteristics of solar cells^19^. Plasma post-treatment technology has been one of the central techniques for thinning the layered materials to a desired thickness and further improve their properties^20^. Precise control of the growth and clear understanding of the mechanism of morphological evolution are the key to obtain a high-quality Bi_2_Se_3_ nanostructure by post-treatment technology. A detailed study about the Bi_2_Se_3_ thin film treated with oxygen plasma helps understand the morphological evolution and thinning mechanism. Various experimental groups have investigated the morphological evolution of the thin film comprising nanosheets by oxygen plasma treatment^17^,^20^,^21^,^22^,^23^,^24^. The atomic-scale mechanisms of graphene etched by oxygen plasma were studied by first-principles molecular dynamics calculations combined with rare events sampling techniques^25^. Although a lot of work has been done on the 2D materials or device surfaces treated with oxygen plasma, the morphological evolution and mechanism of action remain relatively unexplored.

The present study reported the rapid synthesis of high-quality Bi_2_Se_3_ nanosheets by a microwave-assisted technology; these sheets are less toxic, biocompatible, and environmentally friendly. Meanwhile, the study also investigated the use of solution assembly method to prepare Bi_2_Se_3_ thin films from as-prepared few-layer Bi_2_Se_3_ nanoplates dispersed in solvents. Finally, the impact of the plasma treatment on the morphological evolution of Bi_2_Se_3_ thin films thus synthesized was studied.

## Results

**Figure 1:**
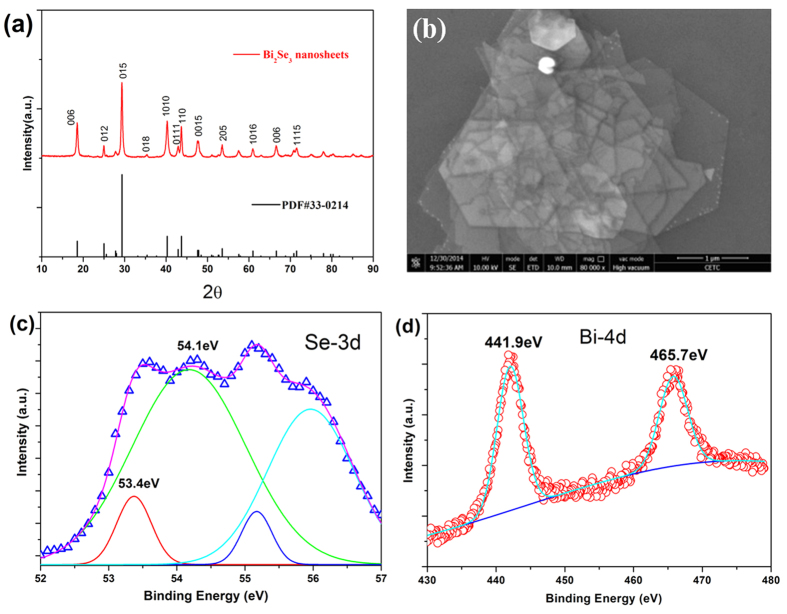
Characterization of the Bi_2_Se_3_ nanosheets. (**a**) Powder X-ray diffraction pattern of Bi_2_Se_3_ nanosheets. (**b**) Typical SEM image of Bi_2_Se_3_ nanosheets (scale bar, 1 μm). (**c**) Detailed photoelectron spectrum of Se 3d. (**d**) Detailed photoelectron spectrum of Bi 4d doublet in Bi_2_Se_3_.

The X-ray diffraction (XRD) pattern of the as-prepared Bi_2_Se_3_ nanosheets is presented in Fig. 1(a). The reflection lines can be readily indexed with the rhombohedral geometry of Bi_2_Se_3_ (JCPDS: 33–0214). No extra peaks were observed for the impurities in the XRD spectrum, suggesting the high purity of the product. The morphology of the as-synthesized Bi_2_Se_3_ nanosheets was characterized using scanning electron microscopy (SEM). Figure 1(b) is a typical low-resolution SEM image of the Bi_2_Se_3_ nanosheets, revealing that the sample consisted of hexagonal nanosheets with an average diameter of 1–2 μm. The x-ray photoelectron spectroscopy (XPS) was used to characterize the surface composition of Bi_2_Se_3_ nanosheets. The Se3d spectrum (Fig. 1(c)) was broad and highly asymmetric, which could be deconvoluted into four peaks, one with a binding energy of 53.4 eV (corresponding to Se 3d5/2) and the other with a binding energy of 54.1 eV (corresponding to Se 3d3/2); the others could be ascribed to oxidation. A high-resolution XPS (Fig. 1d) showed peaks at about 441.9 and 465.7 eV, which corresponded to the reported values for the binding energies of Bi 4d5/2 and Bi 4d7/2, respectively.

**Figure 2:**
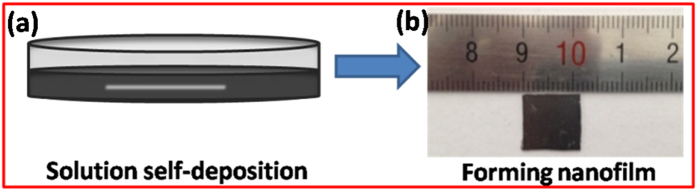
(**a**) Schematic illustration for thin films via evaporation-induced self-assembly process. (**b**) The Bi_2_Se_3_ nanofilm formed.

**Figure 3:**
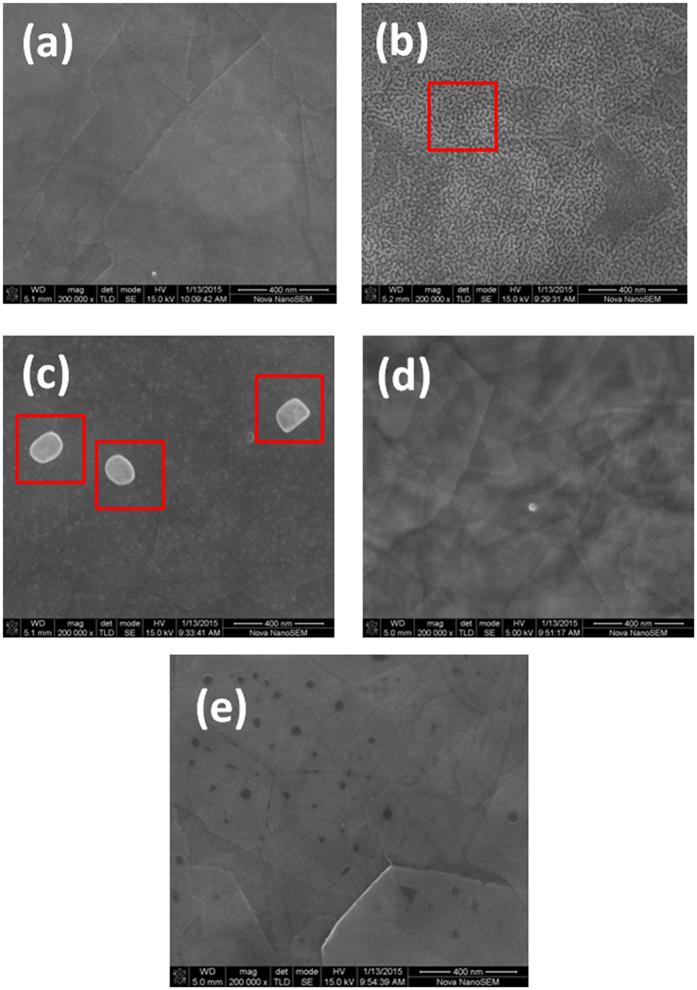
Morphology evolution. SEM image of Bi_2_Se_3_ nanosheets treated by oxygen plasma at different times of 0 s, 10 s, 20 s, 30 s and 50 s, are shown in (**a**–**e**) respectively. Scale bar, 400 nm. The marked areas in (**b**) is the typical area of worm-like structure. The marked areas in (**c**) are the nanoparticles.

**Figure 4:**
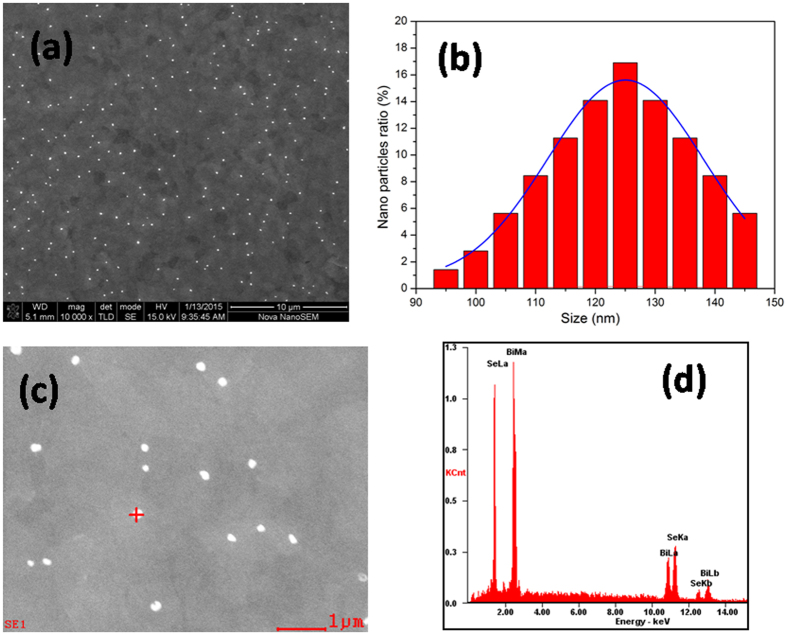
The characteristics of nanoparticles. (**a**)SEM image of nanopaticles formed after 20 s oxygen plasma treatment. (**b**)The lateral size of the nanoparticles from SEM images (**a**) and the Gaussian fitting (blue line) are shown. (**c**)The corresponding elemental compositions of the Bi_2_Se_3_ nanoparticles were determined by energy dispersive spectroscopy, the nanoparticle is marked by the plus sign. (**d**) Corresponding EDS patterns are shown in (**c**).

The thin film was prepared on a silicon wafer via a self-deposition process in Bi_2_Se_3_ nanosheet ink solution. Figure 2(a) shows a schematic illustration for the formation of Bi_2_Se_3_ thin films by the evaporation-induced self-assembly process. 2D Bi_2_Se_3_ nanosheets thus synthesized could be well dispersed in an alcohol solution to form a stable colloidal ink (Fig. 2(b)). The alcohol gradually was evaporated at room temperature, resulting in the Bi_2_Se_3_ nanosheets tiled on the substrate. The effect of the oxygen plasma treatment time on the morphology of the Bi_2_Se_3_ nanosheets was analyzed by SEM images. The SEM images for the samples treated at different times of 0, 10, 20, 30, and 50 s are shown in Fig. 3(a–e), respectively. It was observed that the thin nanosheets were tiled on the silicon wafer one by one, rather than standing vertically on the substrate. The morphology of nanosheets evolved as a worm-like structure when the sample was treated after 10 s. As the treatment time was increased to 20 s, a number of nanoparticles were formed on the surface of nanosheets. It is interesting to note that the sheet was transparent to the electron beam with the increase in the oxygen plasma treatment time to 30 s, indicating that the sheet was very thin. A large number of holes appeared with the increase in time duration of etching to 50 s. This could be ascribed to the fact that the surface layer of Bi_2_Se_3_ nanosheets was oxidized, etched, and then desorbed from the nanosheets. Figure 4(a) further illustrates the surface topography of oxidized Bi_2_Se_3_ nanoparticles. The thin films were acted upon by oxygen plasma for 20 s. It was clearly observed that oxidation led to the formation of well-defined nanostructures. The surface density of nanoparticles was approximately 3.0 × 10^5 ^cm^−2^. The particles were of an average size of 125 nm. The size of the nanoparticles was well described by Gaussian distributions (Fig. 4(b)), indicating that the formation of nanoparticles followed the assembly growth rule. The corresponding elemental compositions of the Bi_2_Se_3_ nanoparticles were determined by energy-dispersive spectroscopy (Fig. 4c,d). The appearance of characteristic peaks corresponded to Bi and Se, and showed that the atomic percentage was slightly higher than the stoichiometry of Bi/Se (=2/3), indicating that Se ions were partly exchanged with O atoms. Hence, it can be concluded that the surface Se ions were replaced by O atoms under the action of oxygen plasma.

**Figure 5:**
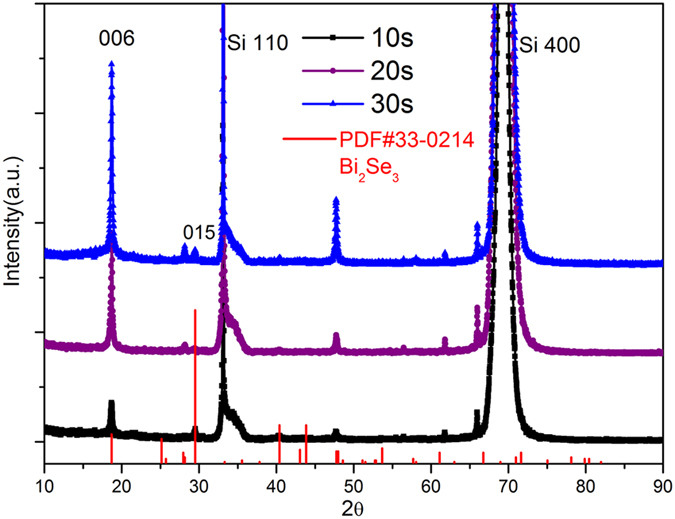
XRD pattern for Bi_2_Se_3_ nanosheet thin film treated by oxygen plasma at different times of 10 s, 20 s, and 30 s, respectively.

Figure 5 shows the XRD patterns of Bi_2_Se_3_ nanosheets treated with oxygen plasma radiation at different times of 10, 20, and 30 s, respectively. Some changes in the positions of peaks could be observed, for instance, the strength of the peaks located at the crystal orientation (006) and (015) changed. The diffraction peaks could be indexed with the paraguanajuatite phase of Bi_2_Se_3_ (PDF#33–0214). This indicated the changes in priority orientation. Compared with the XRD pattern for the Bi_2_Se_3_ powder (Fig. 1a), the observation of (0001) peaks suggested that the nanosheets preferentially lay on the substrate with the same orientation facing upward when self-deposited onto the substrate. As a result, only the plane parallel to the substrate, that is (0001) plane, could contribute to the XRD pattern. Together, the result demonstrated that Bi_2_Se_3_ nanosheets could be assembled into highly uniform thin films with a strong preference in orientation over the large-area substrate using the simple self-deposition process. Since no selenium phase was detected by XRD, it seems likely that the selenium released during the oxidation process left the surface layer of the Bi_2_Se_3_.

## Discussion

**Figure 6:**
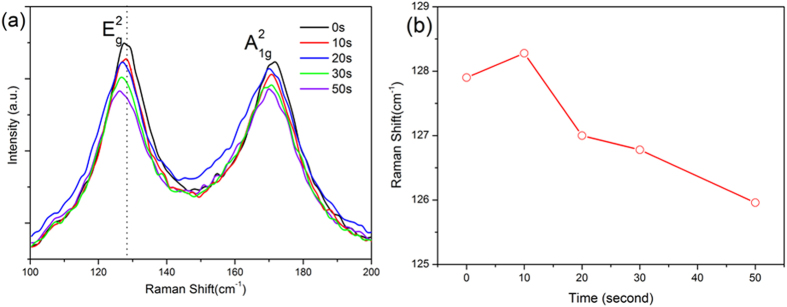
(**a**) Typical Raman spectra of Bi_2_Se_3_ treated by oxygen plasma at different times of 0, 10 s, 20 s, 30 s, and 50 s, respectively. The dot vertical lines highlight the positions of the different vibration modes. (**b**) Positions of the Raman peaks (corresponding to panel (**a**)) as a function of the treatment time.

Although the Bi_2_Se_3_ surface treated with the oxygen plasma can help tune the morphology and size, it may induce plasma damage, including oxidation and etching. The disordering of the Bi_2_Se_3_ surface and oxygen doping may affect the electrical structure and optical properties of Bi_2_Se_3_ thin films. Raman spectra are an efficient tool to investigate the optical characteristics of nanomaterials. It is well known that layered Bi_2_Se_3_ has a rhombohedral structure with the space group of R3-m/D3d5, and is composed of hexagonal close-packed atomic layers periodically arranged along the c-axis. The atomic arrangement can be considered as repeating units, each consisting of five atomic Se1-Bi-Se2-Bi-Se1 layers called quintuple layers, weakly bound by van der Waals forces with a slightly covalent nature. The Raman spectra before and after oxygen plasma etching are shown in Fig. 6(a). It can be clearly seen that two main Raman characteristic peaks assigned to the vibration modes 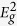 and 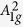, respectively. As the oxygen plasma treatment time was increased from 0–50 s, the peak position of the mode shifted to the low wave (denoted by the vertical dot line), indicating that the inner structure of Bi_2_Se_3_ was not changed by oxygen plasma and mainly the surface of Bi_2_Se_3_ film was oxidized by the oxygen plasma radiation. Se atoms exhibit two different chemical environments. The chemical bonding between Bi and Se2 is of a pure covalent nature, while it is slightly ionic but still covalent in nature between Bi and Se1^11^,^26^,^27^,^28^,^29^,^30^. As for the Bi_2_Se_3_ compounds, O atoms preferentially replace Se at Se2 sites because O atoms at Se2 sites are more stable than the O atoms at Se1 sites, thus resulting in a measurable increase in the values of the lattice parameters of Bi_2_Se_3_. Raman shifting of Bi_2_Se_3_ nanosheets can be ascribed to the change in the center mass due to O occupying the Se site^31^,^32^. However, the crystalline structures and lattice dynamics of Bi_2_Se_3_ are changed, eventually leading to the high-frequency modes and a slight Raman shift and broadening under highly nonequilibrium conditions. This can be ascribed mainly to the rapid oxidation of the surface and morphological evolution. The Raman shifts of the vibrational modes with the increase in oxygen plasma treatment time are summarized in Fig. 6(b). The mode displayed a blue shift when the treatment time was increased. The vibrational mode is sensitive to defects because the interlayer van der Waals interactions influence the effective restoring forces acting on Bi and Se atoms. This effect can be more obvious on the out-of-plane mode because the defect causes a change in not only the restoring forces but also the thickness^30^.

**Figure 7:**
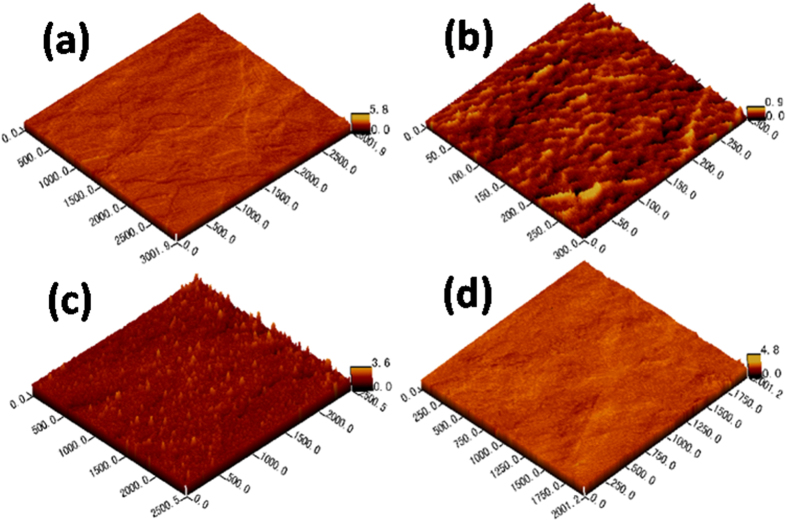
AFM images. The AFM measurement was performed on a local scanning area for the samples treated by oxygen plasma at different times of 0 s, 10 s, 20 s, and 30 s, are shown in (**a**–**d**) respectively.

The atomic force microscopy images of the samples are shown in Fig. 7(a–d). This indicates that oxygen plasma can provide energy to change the morphology of Bi_2_Se_3_ nanomaterial. After the plasma pretreatment from 10–20 s, the surface of Bi_2_Se_3_ film evolved from the worm-like structure to particles. The highly uniform thin film was formed on further increasing the plasma treatment time, which is consistent with the observed SEM results. The root-mean-square (RMS) surface roughness values were extracted from AFM surface images. The RMS decreased from 77% (Fig. 7(a)) to 93% (Fig. 7(d)), indicating considerable improvement compared with the smoothness of self-deposition Bi_2_Se_3_ thin film without the plasma treatment.

To clearly understand the mechanism behind the evolution of the Bi_2_Se_3_ nanosheets acted upon by the oxygen plasma, the following important processes need to be considered:

### Formation of Bi_2_Se_3_ oxide layer

A native bismuth oxide is quickly formed on the surface of Bi_2_Se_3_ thin film. During the oxygen plasma etching process under highly nonequilibrium conditions, O atoms preferentially replace Se atoms on the surface, since inner Se atomic sites are more stable than the surface sites of Se atoms, resulting in the formation of oxygen-doped Bi_2_Se_3_ surface layer.

### Regrowth process

The Bi_2_Se_3_ nanosheets undergo a self-assembling regrowth process under the action of the high-energy oxygen plasma. The morphological evolution of Bi_2_Se_3_ nanosheets is the combined effect of heating and etching of oxygen plasma. The nanoparticle formation is mainly controlled through the migration of adatoms/nanoclusters induced by thermal energy and driving force from the strain between the Bi_2_Se_3_ thin film and the surface oxide layer. The Bi_2_Se_3_ thin film beneath the bismuth oxide layer plays the role of a wetting layer during the regrowth process. Under the continuous action of oxygen plasma, the atoms in the wetting layer or the surface nanoclusters can gain more energy to overcome the strain energy barrier to reach the nucleation centers and regrow. This can be ascribed to the increase in the size of nanoparticle. A previous study showed that the strain energy between the edge of nanoparticles and the wetting layer increases with the growth of nanoparticles, preventing the atoms from diffusing from the wetting layer to the surface of nanoparticles^33^.

### The electrostatic interaction and etching mechanism

Terashima *et al.* showed that the enhancing local electrical field around the vicinity of the boron site on the surface of diamond appears to contribute to the formation of nanostructures during the oxygen plasma treatment process^34^. It is reasonable to imagine that under an energetic oxygen ion attack, the surface of nanosheets can be quickly enriched with electrons. An oxygen-doped Bi_2_Se_3_ surface can enhance the electron emission compared with the intrinsic topological insulator Bi_2_Se_3_. The resulting local electrical field around the surface of the oxygen sites on the Bi_2_Se_3_ surface appears to contribute to the formation of fine nanoparticles during the oxygen plasma etching. Once the nanoparticles are formed, the electrostatic repulsion between nanoparticles and the adjacent quintuple layers can be larger than the van der Waals forces, resulting in the separation of nanoparticles from the Bi_2_Se_3_ matrix. Generally, the topmost layer of Bi_2_Se_3_ sheets is oxidized quickly. The formation of oxides on the surface of nanosheets inhibits further oxidation in the inner layer of the Bi_2_Se_3_ sheets. The peeled-off Bi_2_Se_3_ layer can assemble again due to the action of the high-energy oxygen ion. The particles are formed under the continuous bombardment of the highly energetic oxygen ions. The final nanoparticles are separated from the Bi_2_Se_3_ matrix layer. Thus, in the present case, only the topmost layer was affected. The XRD results and Raman spectra prove this speculation. It opens the possibility of engineering Bi_2_Se_3_ nanostructure and thinning the 2D materials via oxygen plasma treatment.

In conclusion, in this study, Bi_2_Se_3_ nanosheets were successfully synthesized by a microwave-assisted approach in the presence of polyvinylpyrrolidone at a temperature of 180 °C for 2 h. The structure and morphology of the obtained products were characterized by XRD, SEM, XPS, and Raman spectroscopy. The thin film was prepared on a silicon wafer via a self-deposition process in Bi_2_Se_3_ nanosheet ink solution according to the evaporation-induced self-assembly method. The highly uniform Bi_2_Se_3_ nanoparticles could be formed by controlling the oxygen plasma treatment time. Several important processes can result in the morphological evolution and thinning of Bi_2_Se_3_ nanosheets: (1) formation of Bi_2_Se_3_ oxide layer; (2) self-assembly of oxide nanoparticles under the action of the high-energy oxygen plasma; and (3) electrostatic interaction and etching mechanism.

## Methods

The high-quality nanosheets were successfully synthesized by a microwave-assisted approach in the presence of polyvinylpyrrolidone at a temperature of 180 °C for 2 h. 2D Bi_2_Se_3_ nanosheets thus synthesized could be well dispersed in an alcohol solution to form a stable colloidal ink. The thin film was prepared on a silicon wafer via a self-deposition process in a Bi_2_Se_3_ nanosheet ink solution using the evaporation-induced self-assembly method. The alcohol gradually was evaporated at room temperature, resulting in the Bi_2_Se_3_ nanosheets tiled on the substrate. The highly uniform Bi_2_Se_3_ particles could be formed by controlling the oxygen plasma treatment time. After the plasma pretreatment from 10–20 s, the surface of Bi_2_Se_3_ film evolved from the worm-like structure to particles. The highly uniform thin film was formed on further increasing the plasma treatment time. The effect of the oxygen plasma treatment time on the morphology of the Bi_2_Se_3_ nanosheets was analyzed by SEM images.

### Synthesizing of Bi_2_Se_3_ nanosheets and preparing Bi_2_Se_3_ films

All chemicals were analytical grade and used without further purification. In a typical synthesis of Bi_2_Se_3_ nanosheets, 0.2 mmol Bi(NO_3_)_3_·5H_2_O (0.0970 g), 0.3 mmol NaSeO_3_ (0.0519 g) and 2 mmol PVP (0.2223 g) were dissolved in 20 mL ethylene glycol. The mixture was stirred for 10 min and then heated to 180 ^o^C in a 25 mL three-neck flask equipped with thermal couple and reflux condenser in a heating mantle. After 2 hr, the heating mantle was removed and the mixture was allowed to cool down to room temperature naturally. The mixture was then centrifuged at 10 K rpm for 10 min after the addition of 20 mL isopropanol and 10 mL acetone. The washing steps were repeated for three more times. The supernatant was discarded and the solid was dispersed in another 40 mL alcohol. Using the colloidal Bi_2_Se_3_ nanoplates ink, a uniform thin film of Bi_2_Se_3_ can be readily prepared on the silicon substrate by evaporating self assembling process. The (100) intrinsic silicon has been carefully cleaned in a sonicating bath warm acetone (50 °C) for an hour to remove the organic pollutant. The surface oxide layer in the silicon does not remove.

### Plasma treatment

Plasma surface treatment of Bi_2_Se_3_ films was carried out in a plasma cleaner system (Y2008-5C, China). Control samples were not plasma treated. Plasma was created with an inductively coupled RF generator operating at a frequency of 40 kHz with a power 150 W. Flow rates of oxygen were independently controlled and are fixed as 20 sccm. Oxygen pressure was fixed as 5 mTorr. The treatment, temperature of the sample in 273 K.

### Characterization

Characterizations were carried out using scanning electron microscopy (SEM, JEOL JSM-6700F FE-SEM) with energy dispersive spectroscopy (EDAX), X-ray diffraction (XRD, Pan alytical X’Pert Pro X-ray Powder Diffractometer), atomic force microscopy (AFM, Micronano Scanning Probe Microscope), X-Ray Photoelectron Spectroscopy (XPS, PHI 5300), UV-Vis-NIR spectroscopy (Shimadzu 3100 PC). Raman spectroscopy is performed under excitation of a 632 nm laser (15 mW) and a scanning wave number range from 300 cm^−1^ to 100 cm^−1^ in the backscattering configuration.

## Additional Information

**How to cite this article**: Jia, G. *et al.* Morphological evolution of self-deposition Bi_2_Se_3_ nanosheets by oxygen plasma treatment. *Sci. Rep.* **6**, 22191; doi: 10.1038/srep22191 (2016).
